# Classification success of salivary interleukin-1β in periodontitis grading with artificial intelligence models: a cross-sectional observational study

**DOI:** 10.1590/1678-7757-2024-0580

**Published:** 2025-08-08

**Authors:** Erensu UZAR, Ihsan PENCE, Melike Siseci CESMELI, Zuhal YETKIN AY

**Affiliations:** 1 Süleyman Demirel University Faculty of Dentistry Department of Periodontology Isparta Turkey Süleyman Demirel University, Faculty of Dentistry, Department of Periodontology, Isparta, Turkey.; 2 Burdur Mehmet Akif Ersoy University Bucak Faculty of Computer and Informatics Department of Software Engineering Burdur Turkey Burdur Mehmet Akif Ersoy University, Bucak Faculty of Computer and Informatics, Department of Software Engineering, Burdur, Turkey.; 3 Antalya Bilim University Faculty of Dentistry Department of Periodontology Antalya Turkey Antalya Bilim University, Faculty of Dentistry, Department of Periodontology, Antalya, Turkey.

**Keywords:** Artificial intelligence, Biomarkers, Classification, Periodontitis

## Abstract

**Objectives:**

Finding certain biomarkers and threshold values of periodontitis and incorporating them into classifications can further highlight its impact on systemic health. This cross-sectional observational study aims to evaluate the efficacy of some biomarkers in grading periodontitis using artificial intelligence (AI) models.

**Methodology:**

AI models were developed to automatically classify periodontal status (N=240) and grades in periodontitis patients (n=120) using Python based on sociodemographic, anthropometric, clinical, radiological, and biochemical attributes. A total of 35 serum levels of whole blood attributes (white blood cell (WBC), platelet, erythrocyte, neutrophil, lymphocyte counts, and mean platelet volume), lipid profile [triglycerides; high-, low-, and very low-density lipoproteins (HDL, LDL, VLDL), and total cholesterol levels], salivary and serum interleukin (IL)-1β and matrix metalloproteinase (MMP)-8 levels), and 11 other attributes were used in the current classification.

**Results:**

In total, 23 out of 46 attributes achieved a 0.967 classification accuracy, whereas nine, a 0.858 classification accuracy. Attributes such as WBC, serum IL- 1β, triglyceride/HDL ratio, neutrophil/lymphocyte ratio, and HDL were instrumental in periodontal status classification. HDL, LDL, neutrophil/lymphocyte ratio, total cholesterol, salivary IL-1β, and MMP-8 were key attributes in grading.

**Conclusions:**

AI models showed significant classification accuracy, particularly with serum and salivary IL-1β levels and other blood parameters, underscoring the potential of these biomarkers, which could be integrated into the current classification.

## Graphical abstract

**Figure:**
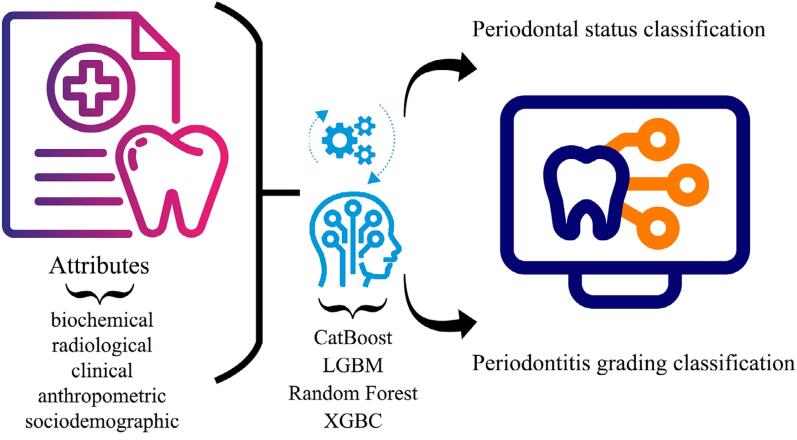

## Introduction

Clinical measurements such as pocket depth (PD), clinical attachment loss (CAL), and bleeding on probing (BOP), along with radiological examinations, are commonly used in the diagnosis of periodontal diseases.^1^ Despite their limitations,^2-5^ periodontal probes (for PD and CAL) and radiographic evaluations remain the most widely used techniques to determine the presence and severity of periodontal disease.⁶ Accurately classifying periodontal diseases and conditions based on clinical and radiographic records is essential for appropriate treatment planning and disease management.

In 2017, the American Academy of Periodontology and the European Federation of Periodontology developed an updated classification to diagnose periodontal diseases, treatment protocols, and inter-professional consensus due to epidemiologic studies. The new classification defines clinical gingival health and gingivitis in intact and reduced periodontia and deems periodontitis as a multidimensional disease, determining severity and progression by a staging and grading system that considers past disease experience and treatment complexity.^7,8^ It also classifies patients based on complicating variables such as CAL, % alveolar bone loss, and PD. Assessing periodontitis progression evaluates factors such as plaque levels, smoking status, metabolic control in diabetes (HbA1c), and high-sensitivity C- reactive protein (hs-CRP) levels. Grading reflects the rate of progression, treatment response, and overall health impact. Furthermore, Determining biomarkers and threshold values in saliva, serum, and gingival crevicular fluid (GCF) can highlight the effect of periodontitis on systemic health.^9^

Advances in technology and the widespread use of diagnostic assays in dentistry and medicine (particularly those with body fluids such as saliva, serum, and GCF) may greatly enhance treatment planning and patient monitoring. Although defining a comprehensive diagnostic set combining clinical, radiological, and laboratory tools remains challenging, it configures a critical future goal. Integrating additional biomarkers is crucial for personalized dentistry applications, which are increasingly important today. In this context, clinical and radiological evaluations should include biochemical and immunological tests for early and accurate disease diagnosis.^10^

Research has explored new diagnostic parameters, such as inflammatory biomarkers. Future validated biomarkers should enhance diagnostic precision in the early detection of periodontitis and offer additional insights to assess its grade.^6^ Salivary analytes, such as interleukin (IL)-1β, IL-6, matrix metalloproteinase (MMP)-8, macrophage inflammatory protein-1α, and prostaglandin E2, are valuable in assessing the prevalence of sites with BOP or increased PD. These findings indicate that salivary profiles are highly accurate in detecting even the smallest presence of the disease and aid the evaluation of periodontitis according to the latest international staging and grading standards.^11^

Dentistry increasingly uses artificial intelligence (AI) for record-keeping, data analysis, disease diagnosis, risk prediction, screening, and education.^12^ Integrating AI into clinical decision-making can significantly enhance diagnostic accuracy and efficiency. AI reduces human error and operator variability by processing large and complex datasets, such as salivary and serum biomarkers. AI-based decision support systems also provide reliable second opinions by detecting subtle patterns and correlations conventional methods may miss, aiding in the early detection and accurate classification of periodontitis. Additionally, AI contributes to standardizing diagnostic protocols, promoting consistency across clinicians and settings.

This study aimed to evaluate the diagnostic accuracy of determining periodontal status and grade in patients with periodontitis by adding clinical, sociodemographic, anthropometric, and biochemical attributes to currently used ones via the most up-to-date AI models. With these models, we aimed to test the hypothesis that no difference in diagnostic accuracy exists between additional clinical, sociodemographic, anthropometric, and biochemical attributes and only using clinical and radiological attributes in AI models.

## Methodology

This study was approved by the Clinical Research Ethics Committee of Suleyman Demirel University Faculty of Medicine (date: 10.03.2022, decision number: 6/73). From April 2022 to May 2023, 240 voluntary participants were recruited from the patients who applied to Süleyman Demirel University Faculty of Dentistry Periodontology Clinic for periodontal treatment and who met the conditions for participation in the study. The 2013 Helsinki Declaration principles were followed in this study.

### Inclusion and exclusion criteria

Volunteers who were aged from 18 to 65 years and who have given their written informed consent after fully understanding the study procedures were included in this study. Exclusion criteria included individuals who had received periodontal treatment in the previous six months, were pregnant or nursing, had psychological or physical impairments, engaged in substance abuse, suffered from significant malocclusion, had acute dental issues such as root-canal infections, pericoronitis or abscesses, had dental caries that required emergent treatment, received a diagnosis of malignant diseases, or had taken medications such as anti-inflammatory drugs or antibiotics that might influence their periodontal health within the last three months.

### Study groups

The sample size for the study was determined by G*Power v3 1.9.2 under a 95% confidence interval, 80% statistical power (with a 0.08 Type II error probability), and a 5% error margin (α: 0.05, β: 0.20). According to these parameters, this study required a total of 240 participants, who were divided into 30-individual groups.

Once the number of participants per group was established, the first 30 individuals who met the diagnostic criteria for each group and the participation criteria in this study and who visited our clinic from April 2022 to May 2023 were assigned to the corresponding groups. No additional participants were added after the groups were formed.

The diagnosis and classification of study participants were established based on the current classification.^7,8^

The study groups were delineated as follows:

1. Clinical gingival health (intact periodontium - Group 1, reduced periodontium - Group 2),
2. Gingivitis (intact periodontium - Group 3, reduced periodontium - Group 4),
3. Periodontitis (Stage I - Group 5, Stage II - Group 6, Stage III - Group 7, Stage IV - Group 8)

Patients with periodontitis were categorized as Grade A, B, and C.

### Clinical periodontal parameters

Gingival index (GI)^13^ and plaque index (PI)^14^ were recorded regarding the mesiobuccal, midbuccal, distobuccal, and mesiolingual/palatal sites of each tooth. BOP^15^, CAL, and PD scores were obtained for the mesiobuccal, mid-buccal, distobuccal, mesiolingual, mid-lingual, and distolingual sites using a Williams periodontal probe (Hu-Friedy Manufacturing Corp., Chicago, IL, USA.). PD and CAL measurements were rounded to the nearest whole mm. BOP percentage was calculated as the number of teeth showing any bleeding.^15^ BOP scores from those six sites were used to calculate the periodontal inflamed (PISA) and periodontal epithelial surface area (PESA) scores.^16^ For each tooth, PESA was calculated using the CAL and recession area and PISA was obtained from PESA by multiplying it by the number of sites with BOP. The sum of individual PISA values results in the full-mouth PISA value in square mm^2^. GI, PI, BOP%, PD, CAL, PISA, and PESA were used as clinical attributes.

Clinical, sociodemographic, anthropometric, components of medical and dental anamnesis (Table 1), and biochemical attributes were added to the attributes in the classification: smoking status: former smoker, <10/day, ≥10/day, and never smoked, clinical attributes: maximum PD, maximum CAL, percentage BOP; radiographic attributes: bone loss pattern (horizontal/vertical), %bone loss (coronal third (<15%), coronal third (15% to 33%) extending to mid-third of root and beyond), number of teeth lost due to periodontitis, presence and extent of furcation involvement, disease distribution (localized, generalized/molar-incisor pattern), bone loss %/age, cause of tooth loss; and biochemical attributes: HbA1c and hs- CRP.

**Table 1:** Descriptive statistics of the study population BMI

Continuous Variables		Mean±SE	Minimum	Maximum
Age		37.70±0.84	18	64
Waist/hip		0.82±0.01	0.59	1.28
BMI		25.76±0.32	15.57	44.96
Categorical Variables		Frequency	Percent	
Gender	Female	126	52.5%	
	Male	114	47.5%	
Education level	Literate	2	0.8%	
	Primary	44	18.3%	
	Secondary	75	31.3%	
	Undergraduate	99	41.3%	
	Higher	20	8.3%	
Systemic disease presence	Yes	174	72.5%	
	No	66	27.5%	
Smoking	Former smoker	36	15%	
	<10 cigarettes/day	27	11.3%	
	≥10 cigarettes/day	65	27.1%	
	Never smoker	112	46.7%	
Tooth brushing frequency	1 time per day	66	27.5%	
	2 times per day	115	47.9%	
	Irregular	55	22.9%	
	Never	4	1.7%	
Use of interproximal cleaning devices	dental floss	69	28.8%	
	Interdental brush	7	2.9%	
	None	164	68.3%	
Monthly income	0-266 USD	96	0,4	
	266-532 USD	108	0,45	
	532-1064 USD	23	9.6%	
	1064 USD and above	13	5.4%	
Frequency of visiting the dentist	Irregular	13	5.4%	
	Every year	64	26.7%	
	When needed	163	67.9%	
Cause of tooth loss	Caries related	96	0,4	
	Periodontitis related	9	3.8%	
	none	1	0.4%	
Systemic drug use	No	174	72.5%	
	Yes	66	27.5%	

BMI: body mass index, USD: US Dollar.

The sociodemographic attributes in this study included age, gender, education level, and monthly income; the anthropometric ones, included weight, height, waist/hip ratio, and body mass index; medical anamnesis ones, systemic diseases, systemic diseases in the family, and the usage of drugs due to systemic diseases; and dental anamnesis ones, the frequency of tooth brushing, the frequency of dental visits, the use of interproximal cleaning devices, and the cause of tooth loss (caries or periodontal).

White blood cell (WBC), neutrophil (NE), lymphocyte, platelet-erythrocyte counts, mean platelet volume (MPV), HbA1c, triglyceride, high, low, very low-density lipoprotein cholesterol (HDL-C, LDL-C, VLDL-C), and total cholesterol (CHOL) levels were determined in serum. Moreover, serum and salivary IL-1β and MMP-8 levels were determined by enzyme-linked immunosorbent assay. Unstimulated whole-mouth saliva samples were centrifuged at 4000× g for 10 min to remove particles, then stored in the early morning at −80 °C until analysis.

All of the attributes above were also used in the AI analyses.

### AI analysis

This study first evaluated the clinical and radiographic attributes in the current classification (n=11) for periodontal status and grading success in patients with periodontitis. After that, a total of 46 attributes were analyzed in conjunction with their success in periodontal status classification and grading in AI models.

Extreme gradient boosting classifier (XGBC), random forest, light gradient boosting machine (LGBM), and categorical boosting (CatBoost) AI models were created using Python software with the clinical, radiological, and biochemical (whole blood, lipid profile, salivary and serum IL-1β and MMP-8) features of 240 patients. A 10-fold cross-validation was applied to ensure that each observation was used for training and testing across iterations. Cross-validation is particularly important when working with a limited dataset as it maximizes data utility, reduces the risk of overfitting, and increases the generalizability of the findings by providing a more robust and reliable estimate of model performance.^17^

XGBC implements a gradient-boosting concept that combines an ensemble of weak learners with a strong learner to improve classification performance. XGBC aims to control overfitting and minimize complexity, leading to better classification results. This computationally fast method determines the errors of prior models and combines them to find the target result. Rather than conventional gradient boosting, XGBC achieves its goal by minimizing training loss and controlling model complexity to avoid over-fitting. It calculates pseudo-residuals for each iteration.^18^

Random forest (a versatile and successful machine learning algorithm) combines multiple randomized decision trees and averages their predictions. This supervised learning method samples data fractions, grows a randomized tree predictor on each piece, and then combines them. Random forests can be applied to various prediction problems, analyze small sample sizes and high-dimensional feature spaces, and can be easily parallelized. It uses techniques such as bagging and the CART-split criterion. Variable importance can be ranked by measures such as mean decrease impurity and mean decrease accuracy. Although other ensemble methods exist (such as gradient tree boosting), random forests offer simple but fundamental ideas for developing state-of-the-art algorithms.^19^

LGBM (a gradient-boosting framework) differs from other algorithms such as XGBC. It uses a “best-first” strategy that splits the node that maximizes the drop in the loss function, leading to “lop-sided” trees, whereas XGBC grows trees to a pre-specified depth. It also has “exclusive feature bundling,” collapsing sparse descriptors into one to increase computational efficiency and information content. LGBM computes faster, uses less memory than XGBC, and provides a method to estimate prediction intervals using a quantile loss function. LGBM delivers prediction accuracies that are comparable to single-task deep neural networks.^20^

CatBoost (a gradient-boosting toolkit) introduces algorithmic techniques to address prediction shifts caused by target leakage. It implements ordered boosting, an alternative to classic algorithms, and incorporates an algorithm to process categorical features. To convert categories into numerical values, CatBoost employs target statistics, estimating the expected target value within each category. To prevent target leakage, CatBoost uses an ordering principle inspired by online learning that introduces a random permutation of training examples, ensuring that target statistics for each example rely solely on the “observed history.” CatBoost operates with two boosting modes: ordered and plain. The ordered boosting mode constructs supporting models, with each model being learned by only the preceding examples in the permutation. The plain boosting mode represents the standard gradient-boosted decision trees algorithm with ordered target statistics. CatBoost leverages combinations of categorical features to capture high-order dependencies. To enhance speed and mitigate overfitting, it uses oblivious decision trees. Furthermore, CatBoost includes a Bayesian bootstrap procedure designed to subsample the dataset in each iteration.^21,22^

All classifiers are based on decision-tree ensembles but differ in how they construct trees, regularize them, and handle data. Random forest builds many full-depth trees on bootstrap samples and averages their votes (bagging). It reduces variance but sometimes underperforming when subtle patterns exist). XGBC and LGBM implement gradient boosting, sequentially fitting trees to residuals: XGBC grows trees level-wise with L1/L2 penalties to curb overfitting, whereas LGBM grows leaf-wise and uses histogram-based splits for faster training on large feature sets but at the risk of deeper, over-specialized trees. The CatBoost ordered boosting and built-in categorical encoding prevent target leakage and minimize preprocessing, offering robustness on mixed data without extensive hyperparameter tuning. These differences in ensemble strategy, regularization mechanisms, tree-building heuristics, and categorical support explain their strengths in handling complex, small-to-medium clinical datasets.

These models classified patients into eight subgroups considering periodontal status (Group 1-8). Periodontitis (A-C) was graded automatically with clinical, radiologic, and biochemical features of 120 individuals of this patient group diagnosed with periodontitis. Feature selection was performed using XGBC and CatBoost AI methods to increase success rates and determine the attributes that play a role in them. Applying feature selection also evinces the influence of individual biomarkers and factors on periodontitis prediction.

## Statistical analysis

Statistical tests were used to determine the significance of the relations between the prediction values of machine learning algorithms. D’Agostino’s normality test and Q-Q plots are foundational tools to evaluate the normality of target variables. The D’Agostino test is a statistical method that quantifies deviations from normality by analyzing skewness (asymmetry) and kurtosis (tail heaviness). The resulting chi-squared statistic (K^2^) is then used to calculate a *p*-value that formally tests the hypothesis of normality and determines its rejection or retention. Statistically significant values are denoted by *p*-values less than 0.05.^23^ At the same time, such statistically rigorous study, sensitivity to sample size, and inability to pinpoint the source of non-normality limit its standalone use. Furthermore, Q-Q plots visually represent the comparison between observed data quantiles and the theoretical quantiles of a normal distribution. Deviations from a straight line, such as S-curves (tail behavior) or systematic bends (skewness), highlight distributional irregularities, offering intuitive insights into the locations and causes of deviations from normality.

**Figure 1:**
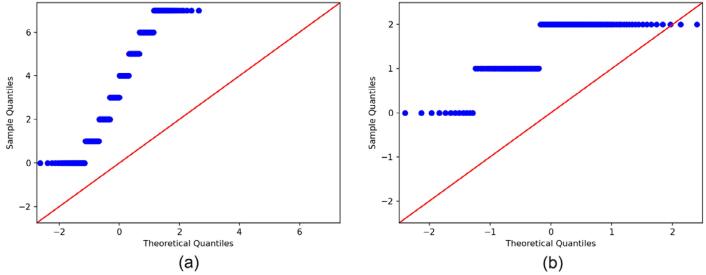
Q-Q plots of targets (a): periodontal status, (b): periodontitis grading

If the assumption of normality is rejected, non-parametric methods such as the Wilcoxon signed-rank test become pertinent. In contrast to parametric tests (e.g., the *t*-test), the Wilcoxon test presupposes no normality and shows resilience to non-normal data. The primary function of this evaluation is to ascertain whether the median of a given sample deviates from a pre-established value or whether paired observations show systematic discrepancies. Symmetry around the median can be tested by ranking absolute differences and analyzing their signs. This approach has effectively analyzed non-normal and ordinal data. The two-sided Wilcoxon signed-rank test is a statistical procedure to evaluate the null hypothesis that the median of the paired differences equals zero. Assuming a valid null hypothesis (H0), the median difference between each pair of observations precisely totals zero. The valid H0 indicates no systematic shifts or effects. The alternative hypothesis (H1) posits that the median difference differs from zero, indicating a consistent change in one direction across the paired samples. Consequently, rejecting the null hypothesis H0 indicates that the observed values systematically differ in the median from their counterparts. The Q-Q plots of periodontal status and periodontitis grading are shown in Figure 1.

The findings, based on the implementation of the D’Agostino’s normality test and Q-Q plots, suggest that the target values are distributed abnormally. In the Q-Q plot (a), the D’Agostino’s normality test yields K^2^=152.12 (p=9.26×10⁻^34^), sample skewness =0.00 (Z=1.01, p=0.3121), and excess kurtosis =–1.2381 (Z=–12.29, p=9.96×10⁻^35^), producing a symmetric, inward-bowed S-shape that reflects no significant asymmetry but highly significant platykurtosis, indicating thin tails than a normal distribution. In contrast, the Q-Q plot (b) also departs from normality (D’Agostino’s K^2^=14.58, p=0.00068) but shows a significant left skew, indicating a tail toward lower values (skew=–0.9047, Z=–3.75, p=0.000176), with no significant kurtosis deviation (excess kurtosis=–0.3578, Z=–0.72, p=0.4742). In summary, the data points strongly deviate from the straight-line pattern expected under normality, indicating that the target values are abnormally distributed. In this study, the Wilcoxon signed-rank test was used due to the non-normal distribution of the target values and the presence of ordinal data.^23^

## Results

**Figure 2:**
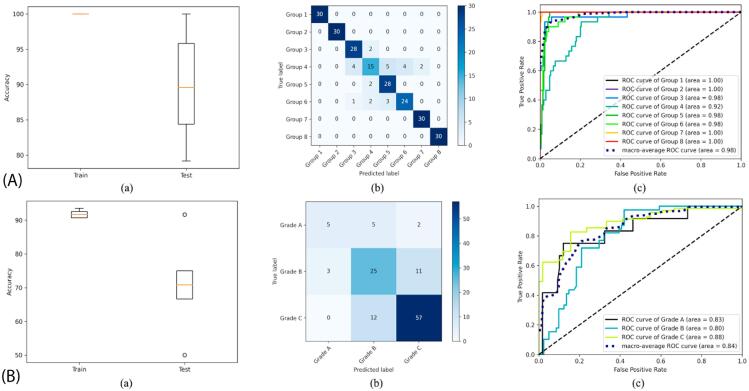
(A): The results of periodontal status classification, (a): box plot, (b): confusion matrix, (c): ROC curve. (B): The results of periodontitis grading classification, (a): box plot, (b): confusion matrix, (c): ROC curve. a) The evaluations were made using the attributes in the current classification.

This study included 240 individuals’ anthropometric, sociodemographic, clinical, radiographic, and biochemical data. Table 2 shows the 10-fold cross-validation results for the machine learning models for periodontal status and grading classification in periodontitis patients, using only 11 attributes in the current classification. Table 2 determined that 11 features [maximum PD, maximum CAL, percentage BOP; radiographic attributes: bone loss pattern (horizontal/vertical), %bone loss (coronal third (<15%), coronal third (15% to 33%), extending to mid-third of root and beyond), number of tooth loss due to periodontitis, presence and extent of furcation involvement, disease distribution (localized, generalized/molar-incisor pattern), bone loss %/age, cause of tooth loss; and biochemical attributes: HbA1c, hs-CRP) showed a 0.896 classification accuracy (CA) in periodontal status classification. Figure 1A (a- c) shows the box plot, confusion matrix, and ROC curve results for periodontal status classification, whereas Figure 1B (a-c), those for grading periodontitis patients. The CA regarding grades in periodontitis patients equaled 0.725 (Table 2). Figure 2(a) shows the features and their importance scores determined due to feature selection for periodontal status classification, whereas Figure 2 (b), those for grading of periodontitis patients.

**Table 2:** Performance of machine learning models for periodontal status and periodontitis grading classification using the attributes in the recent classification.

		Periodontal status						Grade				
Model	Feature Selection	Feature Number	AUC	CA	F1	Precision	Recall	AUC	CA	F1	Precision	Recall
**XGBC**	–	11	0.977	0.867	0.869	0.895	0.867	0.783	0.625	0.612	0.619	0.625
	**XGBC**	3	0.884	0.583	0.539	0.562	0.583	0.775	0.692	0.689	0.723	0.692
		5	0.965	0.833	0.840	0.876	0.833	0.817	0.725	0.716	0.762	0.725
		7	0.972	0.846	0.848	0.881	0.846	0.769	0.683	0.685	0.720	0.683
	**Catboost**	3	0.949	0.779	0.782	0.812	0.779	0.786	0.700	0.692	0.724	0.700
		5	0.972	0.854	0.856	0.885	0.854	0.793	0.625	0.615	0.660	0.625
		7	0.973	0.871	0.873	0.892	0.871	0.795	0.625	0.616	0.631	0.625
**Random Forest**	**–**	11	0.972	0.867	0.872	0.905	0.867	0.788	0.633	0.625	0.649	0.633
	**XGBC**	3	0.875	0.588	0.544	0.552	0.588	0.787	0.683	0.686	0.736	0.683
		5	0.960	0.838	0.843	0.875	0.838	0.834	0.725	0.718	0.750	0.725
		7	0.969	0.867	0.870	0.892	0.867	0.813	0.700	0.688	0.714	0.700
	**Catboost**	3	0.950	0.775	0.777	0.812	0.775	0.806	0.650	0.657	0.688	0.650
		5	0.967	0.871	0.874	0.899	0.871	0.798	0.633	0.629	0.651	0.633
		7	0.972	0.888	0.889	0.910	0.887	0.786	0.667	0.666	0.690	0.667
**LGBM**	**–**	11	0.977	0.850	0.846	0.869	0.850	0.768	0.633	0.629	0.661	0.633
	**XGBC**	3	0.886	0.571	0.519	0.542	0.571	0.820	0.708	0.703	0.757	0.708
		5	0.969	0.838	0.845	0.875	0.838	0.834	0.725	0.714	0.728	0.725
		7	0.977	0.862	0.862	0.884	0.863	0.816	0.692	0.682	0.704	0.692
	**Catboost**	3	0.958	0.779	0.783	0.817	0.779	0.778	0.657	0.658	0.674	0.675
		5	0.975	0.846	0.849	0.872	0.846	0.763	0.608	0.594	0.602	0.608
		7	0.976	0.863	0.865	0.894	0.863	0.767	0.608	0.600	0.616	0.608
**Catboost**	**–**	11	0.982	**0.896**^a)^	0.895	0.915	0.896	0.777	0.642	0.624	0.642	0.642
	**XGBC**	3	0.883	0.592	0.544	0.564	0.592	0.795	0.717	0.717	0.758	0.717
		5	0.974	0.850	0.855	0.884	0.850	0.838	**0.725**^a)^	0.718	0.740	0.725
		7	0.978	0.858	0.856	0.876	0.858	0.811	0.692	0.677	0.706	0.692
	**Catboost**	3	0.960	0.771	0.775	0.814	0.771	0.807	0.717	0.712	0.740	0.717
		5	0.977	0.863	0.865	0.889	0.863	0.794	0.617	0.592	0.601	0.617
		7	0.980	0.871	0.867	0.878	0.871	0.805	0.608	0.586	0.603	0.608

AUC: area under the curve, CA: classification accuracy. ^a)^ Values with high classification accuracy are shown in bold.

The statistical test results of machine learning models for periodontal status and periodontitis grading classification are shown in Table 3.

Table 3 shows the statistical test outcome using the variable h, in which ‘+’ signifies acceptance and ‘-,’ rejection. The *p*-value represents the probability that the *null* hypothesis is valid. The Wilcoxon signed-rank test results in Table 3 evinces no statistically significant difference between the predictions of the machine learning models and patient’s values for periodontal status and grading classification at a *p* = 0.05 significance level. The Wilcoxon signed-rank tests (Table 3) show that, by using only 11 features, no model-feature combinations result in p-values below 0.05 for either periodontal status (p=0.056–1.000) or grading (p=0.078–1.000). The p-values below 0.05 indicate no statistically significant deviation between the cross-validated predictions of the models and the true labels.

**Table 3:** The Wilcoxon signed-rank test results of machine learning models for periodontal status and periodontitis grading classification using the attributes in the current classification.

			The Wilcoxon signed-rank test results			
Model	Feature Selection	Feature Number	Periodontal status		Grade	
			h	*p*	h	*p*
**XGBC**	–	11	+	0.494	+	0.078
	**XGBC**	3	+	0.465	+	0.763
		5	+	0.528	+	0.419
		7	+	0.203	+	0.757
	**Catboost**	3	+	0.235	+	0.098
		5	+	0.096	+	0.054
		7	+	0.197	+	0.349
**Random Forest**	**–**	11	+	0.519	+	0.228
	**XGBC**	3	+	0.582	+	0.670
		5	+	0.748	+	0.317
		7	+	0.632	+	0.305
	**Catboost**	3	+	0.406	+	0.208
		5	+	0.968	+	0.490
		7	+	0.321	+	0.879
**LGBM**	**–**	11	+	0.572	+	0.597
	**XGBC**	3	+	0.361	+	1.000
		5	+	0.983	+	0.096
		7	+	0.141	+	0.168
	**Catboost**	3	+	0.434	+	0.355
		5	+	0.232	+	0.493
		7	+	0.056	+	0.519
**Catboost**	**–**	11	+	0.522	+	0.115
	**XGBC**	3	+	0.158	+	0.887
		5	+	0.385	+	0.419
		7	+	0.455	+	0.196
	**Catboost**	3	+	0.532	+	0.622
		5	+	0.551	+	0.155
				

Figure 2A comprehensively shows model classification of periodontal status into eight groups. Box plot (a) summarizes the distribution of key performance metrics, including medians, quartiles, and potential outliers. This analysis offers insight into the consistency and variability of the model across validation runs or datasets. Confusion matrix (b) details the number of instances correctly or incorrectly classified for each periodontal group, highlighting specific areas in which the model may confuse one status with another. Finally, ROC curve (c) illustrates the trade-off between sensitivity and specificity by plotting the true positive rates against the false positive rates for different classification thresholds. The area under the curve (AUC) constitutes an aggregate measure of the overall diagnostic ability of the model. Collectively, these visualizations underscore the robustness of the model, highlight its classification strengths and weaknesses, and comprehensively depict its capability to distinguish in the eight periodontal status groups. Moreover, Figure 2B shows the corresponding graphs for the periodontitis grading classification.

**Figure 3:**
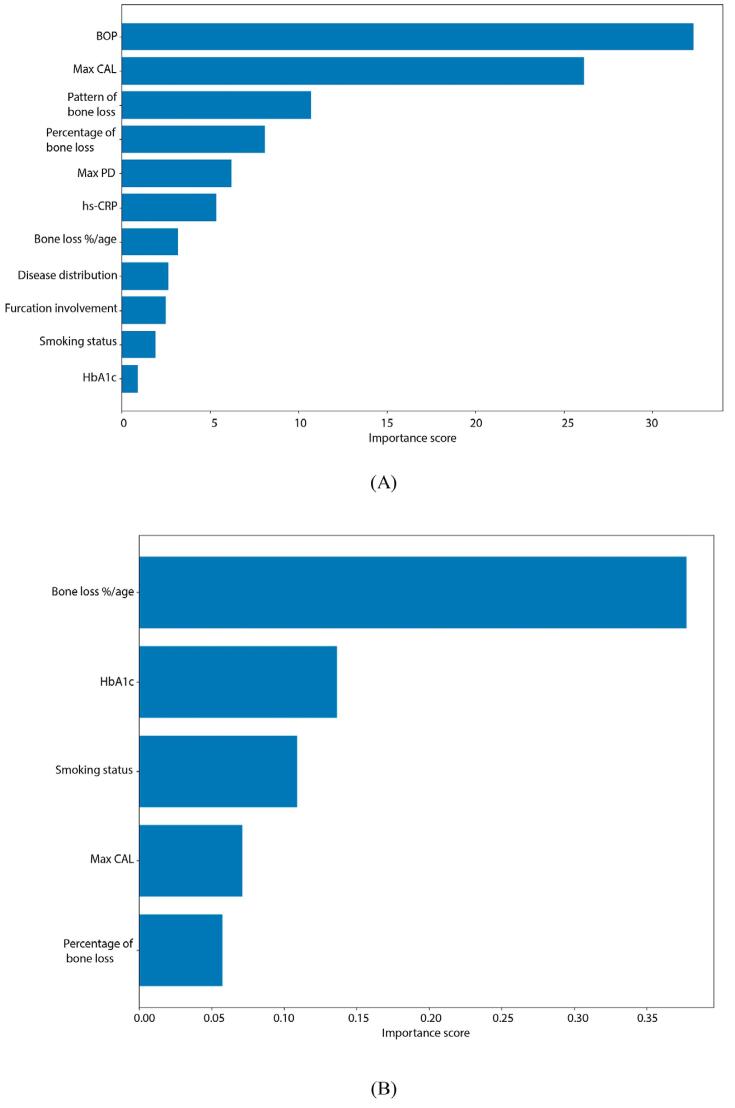
(A): Feature importance scores for periodontal status classification. (B): Feature importance scores for periodontitis grading classification. a) The evaluations were made using the attributes used in the current classification.

Figure 3 highlights the strength of the relation between the factors, the model predictions, and the clinical outcomes. BOP and Max CAL configured as the two most important features for periodontal status classification, whereas bone loss %/age and HbA1c, for periodontitis grading classification.

**Figure 4:**
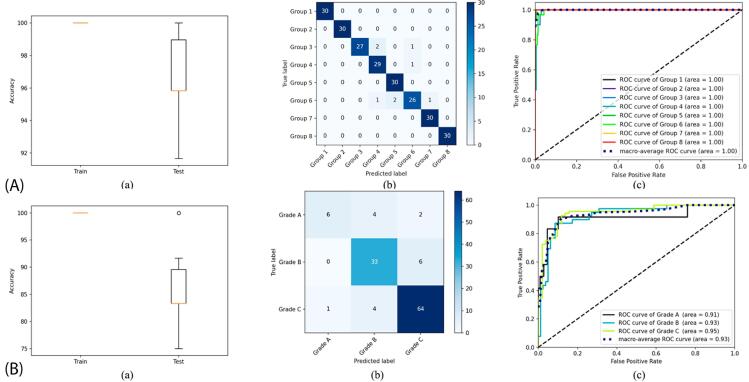
(A): The results of periodontal status classification, (a): box plot, (b) confusion matrix, (c) ROC curve. (B): The results of periodontitis grading classification, (a) box plot, (b) confusion matrix, (c) ROC curve. a) The evaluations were made using additional biochemical attributes.

**Figure 5:**
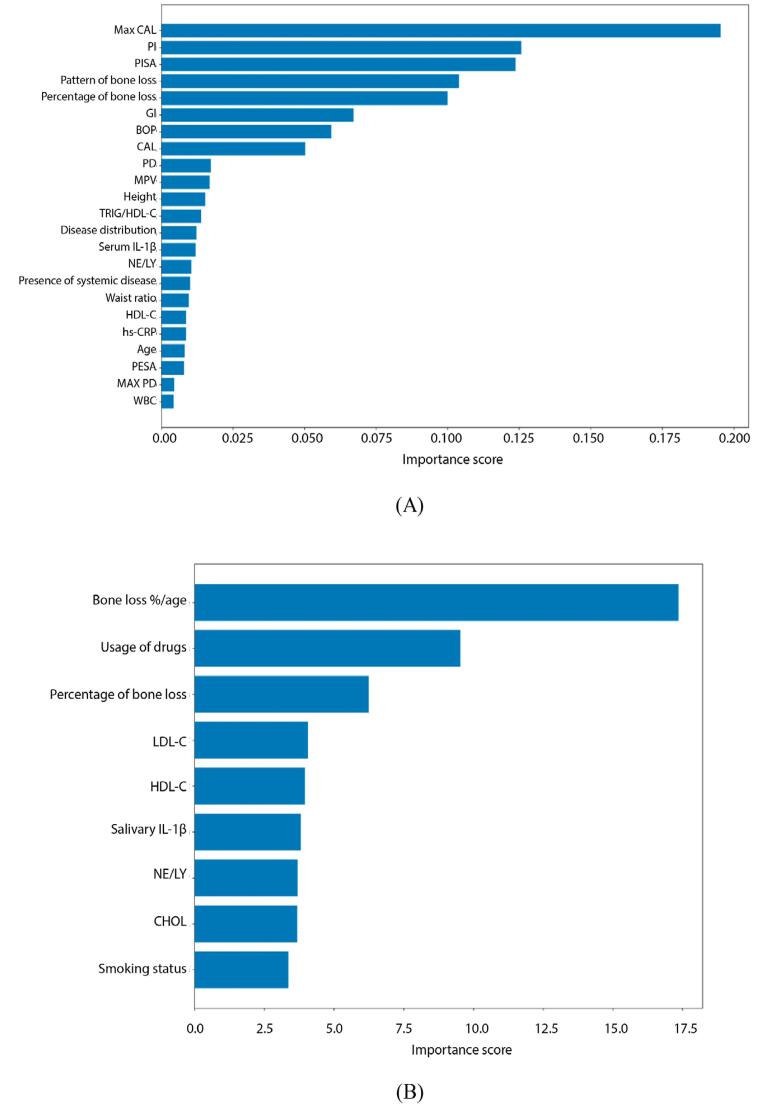
(A): Feature importance scores for periodontal status classification. (B): Feature importance scores for periodontitis grading classification. a) The evaluations were made using additional biochemical attributes.

Table 4 shows the 10-fold cross-validation results for the machine learning models for periodontal status and grading classification in periodontitis patients. It determined that 23 of the 46 features (Max CAL, PI, PISA, pattern and %bone loss, GI, BOP, CAL, PD, MPV, height, Trig/HDL, disease distribution, serum IL-1β, NE/LY, presence of systemic disease, waist, HDL-C, hs-CRP, age, PESA, Max PD, WBC) showed a 0.967 CA in periodontal status classification. Figure 4A (a-c) shows the box plot, confusion matrix, and ROC curve results for periodontal status classification, whereas Figure 5 (a), the features and their importance scores as a result of feature selection for periodontal status classification.

**Table 4:** Performance of machine learning models for periodontal status and periodontitis grading classification using additional sociodemographic, anthropometric, clinical, and biochemical attributes.

		Periodontal status						Grade				
Model	Feature Selection	Feature Number	AUC	CA	F1	Precision	Recall	AUC	CA	F1	Precision	Recall
**XGBC**	**–**	46	0.994	0.938	0.931	0.931	0.938	0.864	0.760	0.749	0.777	0.760
	**XGBC**	6	0.992	0.888	0.884	0.903	0.887	0.876	0.750	0.739	0.779	0.750
		9	0.997	0.917	0.913	0.922	0.917	0.856	0.742	0.727	0.764	0.742
		13	0.997	0.942	0.936	0.939	0.942	0.902	0.800	0.783	0.813	0.800
		23	0.997	0.946	0.938	0.938	0.946	0.933	0.825	0.819	0.850	0.825
		32	0.996	0.946	0.939	0.940	0.946	0.906	0.800	0.789	0.804	0.800
	**Catboost**	6	0.995	0.946	0.946	0.959	0.946	0.889	0.792	0.782	0.802	0.792
		9	0.998	0.933	0.933	0.943	0.933	0.941	0.850	0.836	0.854	0.850
		13	0.997	0.929	0.922	0.928	0.929	0.926	0.817	0.803	0.818	0.817
		23	0.997	0.929	0.924	0.930	0.929	0.919	0.817	0.810	0.843	0.817
		32	0.996	0.938	0.932	0.937	0.938	0.896	0.758	0.751	0.782	0.758
**Random Forest**	**–**	46	0.996	0.929	0.924	0.938	0.929	0.861	0.704	0.675	0.681	0.704
	**XGBC**	6	0.991	0.913	0.910	0.922	0.912	0.902	0.783	0.783	0.831	0.783
		9	0.997	0.929	0.921	0.927	0.929	0.888	0.783	0.783	0.835	0.783
		13	0.998	0.938	0.933	0.944	0.938	0.888	0.783	0.781	0.824	0.783
		23	0.997	0.929	0.923	0.936	0.929	0.902	0.767	0.746	0.759	0.767
		32	0.996	0.929	0.920	0.926	0.929	0.861	0.708	0.699	0.745	0.708
	**Catboost**	6	0.993	0.950	0.952	0.963	0.950	0.884	0.792	0.791	0.821	0.792
		9	0.995	0.933	0.933	0.947	0.933	0.914	0.775	0.770	0.804	0.775
		13	0.995	0.925	0.916	0.921	0.925	0.903	0.750	0.748	0.810	0.750
		23	0.995	0.921	0.912	0.917	0.921	0.871	0.700	0.688	0.729	0.700
		32	0.995	0.933	0.928	0.941	0.933	0.846	0.692	0.685	0.738	0.692
**LGBM**	**–**	46	0.996	0.950	0.942	0.949	0.950	0.772	0.678	0.651	0.656	0.678
	**XGBC**	6	0.989	0.900	0.898	0.916	0.900	0.849	0.725	0.710	0.751	0.725
		9	0.998	0.946	0.940	0.948	0.946	0.824	0.733	0.722	0.746	0.733
		13	0.997	0.942	0.936	0.946	0.942	0.818	0.717	0.688	0.688	0.717
		23	0.997	0.946	0.939	0.945	0.946	0.830	0.717	0.709	0.744	0.717
		32	0.997	0.942	0.935	0.941	0.942	0.825	0.683	0.664	0.673	0.683
	**Catboost**	6	0.996	0.938	0.937	0.948	0.938	0.844	0.733	0.725	0.752	0.733
		9	0.998	0.958	0.956	0.968	0.958	0.870	0.758	0.752	0.779	0.758
		13	0.997	0.938	0.932	0.939	0.938	0.888	0.775	0.751	0.756	0.775
		23	0.997	0.954	0.949	0.952	0.954	0.826	0.708	0.697	0.721	0.708
		32	0.997	0.950	0.943	0.945	0.950	0.803	0.675	0.661	0.676	0.675
**Catboost**	**–**	46	0.998	0.950	0.949	0.957	0.950	0.853	0.694	0.665	0.692	0.694
	**XGBC**	6	0.993	0.908	0.907	0.923	0.908	0.869	0.783	0.782	0.819	0.783
		9	0.998	0.954	0.954	0.963	0.954	0.887	0.808	0.804	0.853	0.808
		13	0.999	0.946	0.946	0.959	0.946	0.896	0.783	0.772	0.816	0.783
		23	0.999	**0.967**	0.965	0.975	0.967	0.891	0.758	0.741	0.789	0.758
		32	0.998	0.954	0.955	0.966	0.954	0.879	0.708	0.683	0.695	0.708
	**Catboost**	6	0.996	0.946	0.945	0.955	0.946	0.889	0.775	0.767	0.781	0.775
		9	0.999	0.954	0.954	0.966	0.954	0.929	**0.858**^a)^	0.847	0.863	0.858
		13	0.999	0.958	0.959	0.971	0.958	0.924	0.800	0.786	0.807	0.800
		23	0.999	0.963	0.961	0.974	0.963	0.876	0.733	0.707	0.719	0.733
		32	0.998	0.950	0.952	0.964	0.950	0.865	0.733	0.704	0.719	0.733

AUC: area under the curve, CA: classification accuracy. ^a)^ Values with high classification accuracy are shown in bold.

Table 5 shows the statistical test results by using the variable h, in which ‘+’ symbol indicates acceptance and ‘-,’ rejection. A similar pattern occurred in the expanded 46-feature set (Table 5), in which most comparisons yielded non-significant *p*-values, substantiating the close alignment with patient values. However, only a few specific configurations showed a significant association with the outcome. The XGBC model, with 13 features for status (*p*=0.027); the CatBoost model, with nine features for grading (*p*=0.004) and 13 features for status (*p*=0.014); the CatBoost model, with 32 features for status (*p*=0.030); and the LGBM model, with 13 features for grading (*p*=0.029 showed statistical significance. These findings suggest the further refinement and calibration of these model/subset pairings. These statistical outcomes show that the AI classifiers, across most feature selections, generate predictions using additional sociodemographic, anthropometric, clinical, and biochemical attributes that are indistinguishable from the true clinical classifications.

**Table 5:** The Wilcoxon signed-rank test results of machine learning models for periodontal status and periodontitis grading classification using additional sociodemographic, anthropometric, clinical, and biochemical attributes.

			The Wilcoxon signed-rank test results			
Model	Feature Selection	Feature Number	Periodontal status		Grade	
			h	*p*	h	*p*
**XGBC**	**–**	46	+	0.171	+	0.333
	**XGBC**	6	+	0.451	+	0.583
		9	+	0.268	+	0.134
		13	-	0.027	+	0.106
		23	+	0.110	+	0.292
		32	+	0.124	+	0.237
	**Catboost**	6	+	0.147	+	0.264
		9	+	0.383	-	0.004
		13	-	0.014	+	0.088
		23	+	0.083	+	0.211
		32	-	0.030	+	0.301
**Random Forest**	**–**	46	+	0.980	+	0.691
	**XGBC**	6	+	0.263	+	0.967
		9	+	0.698	+	0.606
		13	+	0.748	+	0.862
		23	+	0.981	+	0.420
		32	+	0.641	+	0.886
	**Catboost**	6	+	0.338	+	0.397
		9	+	0.812	+	0.413
		13	+	0.542	+	0.763
		23	+	0.615	+	1.000
		32	+	0.893	+	0.975
**LGBM**	**–**	46	+	0.294	+	0.181
	**XGBC**	6	+	1.000	+	0.270
		9	+	0.253	+	0.176
		13	+	0.210	-	0.029
		23	+	0.129	+	0.276
		32	+	0.210	+	0.078
	**Catboost**	6	+	0.662	+	0.312
		9	+	0.145	+	0.489
		13	-	0.042	-	0.032
		23	+	0.203	+	0.162
		32	+	0.159	+	0.224
**Catboost**	**–**	46	+	1.000	+	0.107
	**XGBC**	6	+	0.266	+	1.000
		9	+	0.463	+	0.414
		13	+	0.490	+	0.761
		23	+	0.429	+	0.277
		32	+	0.647	+	0.230
	**Catboost**	6	+	0.913	+	0.773
		9	+	0.463	+	0.122
		13	+	0.565	+	0.518
		23	+	0.903	+	0.080
		32	+	0.516	+	0.157

Table 5 shows the statistical test results of using the variable h, in which ‘+’ indicates acceptance and a ‘-,’ rejection

Figure 4 shows the performance of a periodontal status and periodontitis grading classification model in three ways: a box plot summarizes the consistency and variability of accuracy metric, a confusion matrix shows how often each of the eight groups in the periodontal status classification and the three groups in the periodontitis grading classification received correct or incorrect classifications, and an ROC curve shows the trade-off between true positive and false positive rates, reflecting overall diagnostic accuracy.

Figure 5 highlights the strength of the relation between the factors and the model predictions for periodontal status and grading classification using additional sociodemographic, anthropometric, clinical, and biochemical attributes. Max CAL and PI configured the two most important features for periodontal status classification, whereas bone loss %/age and usage of drugs, for periodontitis grading classification.

According to Table 4, nine features (bone loss %/age, usage of drugs due to systemic diseases, %bone loss, LDL-C, HDL-C, salivary IL-1β, NE/LY, CHOL, and smoking) showed a 0.858 CA in grading classification of periodontitis. While the classification features included WBC, serum IL-1β, Trig/HDL ratio, NE/LY ratio, and HDL, grading included HDL-C, LDL-C, NE/LY ratio, CHOL, and salivary IL-1β, which served no purpose staging classification and periodontitis grading. Figure 5B (a-c) shows the box plot, confusion matrix, and ROC curve results for periodontitis grading classification, whereas Figure 4(b), those for periodontitis grading classification, the features determined due to feature selection, and their importance scores.

According to the other results, in the classification success of periodontal status, the highest CA equaled 0.312 in the analysis using only whole blood, saliva, and serum attributes without clinical and radiographic attributes. Including PISA and PESA obtained a 0.679 CA according to the five best attributes (PISA, PESA, serum MMP-8, salivary IL-1β, and hs- CRP), whereas the CA according to the nine best features (PISA, PESA, serum MMP-8, salivary IL-1β, hs-CRP, HDL-C, MPV, Trig, and NE/LY) totaled 0.677. Analyzing classification success obtained a 0.312 CA only with PISA, a 0.587 one only with PESA, and a 0.671 one with PISA-PESA.

The highest CA for the grading success of patients with periodontitis equaled 0.645 using only whole blood, saliva, and serum attributes without clinical and radiographic attributes. By including PISA and PESA, the CA for the five best features (NE/LY, HDL-C, LDL-C, PISA, and salivary MMP-8) equaled 0.695, whereas that for the nine best attributes (NE/LY, HDL- C, LDL-C, salivary MMP-8, MPV, PISA, PESA, CHOL, and Trig/HDL), 0.703. The grading success CA equaled 0.603 with PISA only, 0.577 with PESA only, and 0.578 with PISA and PESA.

According to the results of periodontal status classification only using clinical and radiographic features the highest success rate equaled 0.954 with CatBoost using all 14 features. As a result of CatBoost feature selection, the success rate increased to 0.958 with the LGBM algorithm by using the best nine features (Max CAL, GI, PISA, CAL, PD, BOP, %bone loss, pattern of bone loss, and PESA). According to the results of periodontitis grading classification, while the success rate equaled 0.592 with the LGBM algorithm using all features, the success rate increased to 0.658 with the CatBoost algorithm with the four best features (bone loss %/age, GI, PI, PISA).

The Wilcoxon signed-rank test assessed the performance of both models, obtaining non- significant results, in line with the high accuracies of these models. During staging, the CatBoost model with the XGBC feature selection with 23 features (CA=0.967) yielded a *p* = 0.429; whereas the CatBoost model with CatBoost feature selection with nine features (CA=0.858), a *p*=0.122 for grading. As both *p*-values exceed 0.05, no evidence exists of a systematic shift between predictions and true labels. Consequently, no discrepancy occurred between the statistical tests and the observed classification accuracies. Overall, the consistently high performance of CatBoost—evinced by non-significant Wilcoxon tests and optimal classification accuracies in staging and grading—is likely attributable to its ordered boosting framework and native target-based encoding of categorical variables. These characteristics mitigate the effects of overfitting and effectively capture the intricate interactions among clinical and biochemical features. Also, the expanded feature set showed that Max CAL and PI dominated status classification. At the same time, bone loss %/age and usage of drugs configured the most critical factors for grading.

## Discussion

This study aimed to evaluate CA in determining periodontal status and grade in periodontitis patients by adding sociodemographic, anthropometric, medical, and dental anamnesis components, biochemical attributes (whole blood parameters, lipid profiles and salivary and serum IL-1β and MMP-8), and features in the current classification.^7^ The chosen AI models obtained a high degree of CA in their classification of periodontal status and grading of periodontitis patients. Moreover, some of the examined blood parameters (HDL-C, VLDL-C, CHOL, LDL-C, Trig/HDL, and hs-CRP, WBC) and salivary and serum IL-1β and MMP-8 contributed to this CA success with higher importance scores.

IL-1β constitutes a pivotal pro-inflammatory cytokine that mediates the host immune response in periodontal disease. It recruits inflammatory cells to periodontal tissues, stimulates osteoclast differentiation, and enhances bone resorption, contributing to tissue destruction and disease progression.^24^ MMP-8 (also known as neutrophil collagenase) plays a crucial role in the degradation of type I collagen, a major component of the periodontal extracellular matrix. Elevated levels of MMP-8 are associated with increased periodontal tissue breakdown and are considered a reliable biomarker for disease activity.^25^ HDL possesses anti-inflammatory properties that benefit periodontal health. It can inhibit the expression of adhesion molecules and cytokines, reducing the inflammatory response. Conversely, periodontitis has been linked to decreased HDL levels, suggesting a bidirectional relation between lipid metabolism and periodontal inflammation.^25^ Incorporating these biomarkers into periodontal disease classification enhances diagnostic precision by reflecting the current inflammatory status and tissue destruction level, facilitating more accurate staging and grading of the disease.

Studies in the literature evaluated radiographs^26-29^ and diagnosed gingivitis by photographs^29^ in comparison to the use of AI in periodontitis staging and grading. However, no other study has a scope similar to ours. In our previous study, several AI models obtained a 97.2% staging and grading success.^30^ Its algorithm optimization was yet to be completed due to the lack of Stage I and II periodontitis patients in its study population. This follow-up study included a sufficient number of individuals in each stage. It also added clinical gingival health and gingivitis groups with intact and reduced periodontium and forwarded research by using additional attributes (e.g., IL-1β, HDL, MMP-8). It obtained a 0.725 CA with only 11 attributes in the current classification (i.e., without adding 35 features).

Our previous study^30^ obtained a 0.986 such value with the random forest tree and k-nearest neighbor algorithm and only 1 Stage I and 3 Stage II patients. This difference regarding CA values between this and previous studies might depend on the AI models/algorithms or periodontal status groups. This study obtained a 100 CA for Stage III and Stage IV and 28 and 24 values for Stage I and II, respectively. Despite its low value in the hierarchy of scientific evidence our clinical observations hinder the distinction between Stage I and II. In some cases, it is impossible to distinguish between gingivitis patients with reduced periodontium and Stage I periodontitis patients. Using 11 attributes regarding gingivitis with reduced periodontium, five of 30 patients received a classification of gingivitis with intact periodontium; five of them Stage I periodontitis; four of them Stage II periodontitis, and four, Stage III periodontitis.

This classification using 23 attributes yielded better results, with only one patient classified as gingivitis with reduced periodontium; two, as Stage I periodontitis; and 30, as Stage II. All of the Stage I periodontitis patients received a 100-CA classification in this study using AI models other than those in our previous study.^30^ This result supported our hypothesis that increasing the number of attributes would improve classification success according to periodontal status.

In addition to smoking/amount of smoking and HbA1c and hs-CRP values (considered in the current classification for grading), whole blood, salivary, and serum attributes evaluated, and the classification and grading success were found to be promising. NE/LY ratio, a potential biomarker for systemic inflammatory response related to periodontitis,^27^ some dyslipidemia indicators (decreased HDL-C, increased Trig, LDL-C, CHOL, Trig/HDL ratio due to periodontitis),^28^ salivary and serum IL-1β and serum MMP-8 levels, which increase with the severity of periodontitis,^29^ may constitute biomarkers/attributes that can be included in the classification of periodontal status and grading of periodontitis. However, evaluated our findings showed the increased classification and grading success of only whole blood, salivary, and serum attributes with the clinical and radiographic features. Although the salivary and serum attributes in our study had a high classification and grading success, they only support the recommended clinical and radiographic attributes to classify, grade, and evaluate the effect of periodontal status on systemic inflammation. Evaluating the importance scores showed that BOP and max CAL obtained the highest periodontal status classification score, whereas bone loss%/age and HbA1c, that for periodontitis patients grading. Nevertheless, whole-mouth saliva comprises salivary secretions, GCF, and epithelial cells. Therefore, the salivary levels of IL-1β and MMP-8 in this study may partly reflect contributions from GCF. This potential overlap configures a limitation to finding interpretation.

Using 23 attributes obtained the highest importance scores for periodontal status classification for max CAL, PI, and PISA; for grading bone loss%/age, using drugs for systemic diseases and bone loss% showed the highest importance scores in the nine attributes to grade periodontitis patients. Although it fails to show the highest one, salivary IL- 1β obtained a higher score than smoking status, which the current classification uses to grade periodontitis patients.

Although the AI in our study is up-to-date, the fact that the data we evaluated lacked the ideal size can configure a limitation of our study. However, our research offers valuable findings for further studies for clinical use with a larger sample assessing the external reliability and validation of the models (assuming that our study is a pioneering study on this subject).

Besides, using all these parameters in real-life scenarios is difficult. This study proposed an AI model that will help dentists/periodontists make diagnostic decisions with fewer parameters. A system is to be developed with the model in this study. That proposed study enhances reproducibility and clinical applicability in periodontitis classification by detailing the performance of popular machine learning algorithms and employing a feature selection process to pinpoint key biomarkers. This transparency facilitates replication by other researchers and supports future model development and refinement in clinical settings. The benefits and success of the system for real clinical applications will be measured.

## Conclusion

With the high classification and grading accuracy using salivary and serum IL-1β along with other blood-based parameters, this study provides important preliminary evidence for a subset of biomarkers that can effectively classify periodontal status and assess systemic involvement. Although incorporating a broad panel of biomarkers into daily clinical workflows can offer challenges, our AI-based model shows that high diagnostic performance can stem from a reduced set of clinically accessible parameters. This supports the feasibility of implementing such models in real-life settings. A decision-support system based on the model developed in this study is currently under planning to be integrated into user-friendly software platforms for use in dental clinics. These systems could assist clinicians by offering real-time, objective assessments of periodontitis stage and grade, especially in complex cases. Moreover, many of the used biomarkers—such as IL-1β, HDL, and neutrophil/lymphocyte ratio—are measurable by standard blood tests or salivary diagnostics, enhancing the translational potential of our approach. By detailing model performance and key attributes, the study ensures reproducibility and promotes the integration of AI into routine diagnostic protocols, paving the way for more personalized and evidence-based periodontal care.
